# Trends for coronary heart disease and stroke mortality among migrants in England and Wales, 1979–2003: slow declines notable for some groups

**DOI:** 10.1136/hrt.2007.122044

**Published:** 2007-08-09

**Authors:** S Harding, M Rosato, A Teyhan

**Affiliations:** 1 Medical Research Council, Social and Public Health Sciences Unit, Glasgow, UK; 2 Department of Epidemiology and Public Health, Queen’s University Belfast, UK

## Abstract

**Objective:**

To examine trends in coronary heart disease and stroke mortality in migrants to England and Wales.

**Design:**

Cross-sectional.

**Outcome measures:**

Age-standardised and sex-specific death rates and rate ratios 1979–83, 1989–93 and 1999–2003.

**Results:**

Coronary mortality fell among migrants, more so in the second decade than the first. Rate ratios for coronary mortality remained higher for men and women from Scotland, Northern Ireland, Republic of Ireland and South Asia, and lower for men from Jamaica, other Caribbean countries, West Africa, Italy and Spain. Rate ratios increased for men from Jamaica (1979–83: 0.45, 0.40 to 0.50; 1999–2003: 0.81, 0.73 to 0.90), Pakistan (1979–83: 1.14, 1.04 to 1.25; 1999–2003: 1.93, 1.81 to 2.06), Bangladesh (1979–83: 1.36, 1.15 to 1.60; 1999–2003: 2.11, 1.90 to 2.34), Republic of Ireland (1979–1983: 1.18, 1.15 to 1.21; 1999–2003: 1.45, 1.39 to 1.52) and Poland (1979–83: 1.17, 1.09 to 1.25; 1999–2003: 1.97, 1.57 to 2.47), and for women from Jamaica (1979–83: 0.63, 0.52 to 0.77; 1999–2003: 1.23, 1.06 to 1.42) and Pakistan (1979–83: 1.14, 0.88 to 1.47; 1999–2003: 2.45, 2.19 to 2.74), owing to smaller declines in death rates than those born in England and Wales. Rate ratios for stroke mortality remained higher for migrants. As a result of smaller declines, rate ratios increased for men from Pakistan (1979–1983: 0.99, 0.76 to 1.29; 1999–2003: 1.58, 1.35 to 1.85), Scotland (1979–1983: 1.11, 1.04 to 1.19; 1999–2003: 1.30, 1.19 to 1.42) and Republic of Ireland (1979–1983: 1.27, 1.19 to 1.36; 1999–2003: 1.67, 1.52 to 1.84).

**Conclusion:**

For groups with higher mortality than people born in England and Wales, mortality remained higher. Smaller declines led to increasing disparities for some groups and to excess coronary mortality for women from Jamaica. Maximising the coverage of prevention and treatment programmes is critical.

Ethnic-specific trends in death rates provide aetiological clues about how environmental exposures affect susceptibility to disease, as well as how successfully particular conditions are managed. Over the past 30 years in England and Wales, the highest death rates for coronary heart disease have been found in migrants of Indian subcontinent origin and the highest death rates for stroke in migrants of Caribbean and directly African origin.^1^^–^4 The last two groups have had coronary rates that are half that of the national rates. The cause of these patterns is unclear. Both groups have a higher prevalence of diabetes and impaired glucose tolerance and people of African origin more hypertension.^5^ ^6^ Other traditional risk factors, such as smoking or cholesterol levels (lower in Caribbeans), only partially account for differences in cardiovascular risk. In the USA, as a result of the slower decline in coronary death rates among Black Americans than White Americans, Black American women have had higher rates since the mid-1980s and Black American men since 2000.^7^ Poorer access to specialised clinical cardiovascular care and economic disadvantage have contributed to these trends.

Annual death rates for migrant or ethnic groups are not available in Britain. Analysis relies on decennial rates based on information by country of birth from the census and from deaths registered around the time of the census. Between 1970–2 and 1989–92, coronary and stroke mortality declined among migrants from Scotland, Ireland, the Caribbean and South Asia, least for South Asians.^2^ Very little is known about the epidemiology of cardiovascular disease among Southern or Eastern European migrants in the UK. In this paper we examine trends in migrant mortality from coronary heart disease and stroke between 1979 and 2003, in these groups as well as migrants from Southern and Eastern Europe and France.

## METHODS

The Office for National Statistics provided anonymised death records for 1979–83, 1989–93 and 1999–2003, and tabulated population data from the 1981, 1991 and 2001 censuses for England and Wales. Deaths and populations at risk were derived by country of birth and 5-year age groups, with analyses based on those aged 30–69 years only. The age restriction was due to the small number of deaths at younger ages and the poor quality of denominator data at older ages for some groups. Countries of birth were included if definitions of countries were comparable over the three time periods in both the deaths and census data, and if there were at least 20 deaths in each 10-year age group in 1999–2003 and at least one previous time period. An East African group, comprising migrants from Kenya, Malawi, Tanzania, Uganda or Zambia, was separately defined because of the likelihood that this group contained large numbers of people of Indian origin. A group drawn from Western and Southern Africa (termed West Africa in the tables) was defined, comprising migrants from Gambia, Ghana, Sierra Leone, Nigeria, Botswana, Lesotho, Swaziland or Zimbabwe. In the 1981 census tables, Lesotho and Swaziland were not identifiable and were not included in either the populations or deaths for 1979–83. Those from Jamaica were identified separately as, unlike some Caribbean countries, the majority of its population is of West African heritage.

The 9th International Classification of Disease was used to classify deaths occurring between 1979 and 2000 (codes 4100–4149 for coronary heart disease (abbreviated to coronary hereafter) and 4300–4389 for strokes) and the 10th for deaths between 2001 and 2003 (I20–I25 for coronary heart disease, I60–I69 for strokes). Trends in absolute mortality were assessed using directly standardised rates, adjusted to the European standard population 2000. Trends in relative mortality were assessed using rate ratios derived from the standardised rates, with the rate for those born in England and Wales as baseline. In the text, excess mortality refers to rate ratios statistically significantly higher than 1.00, significant differences between rate ratios and rates refer to p<0.05, the first decade refers to the time between 1989–93 and 1979–83, and the second decade to the time between 1999–2003 and 1989–93.

## RESULTS

Table 1 shows the populations and coronary death rates for the three time periods. The populations increased for most groups, notable exceptions being for men born in Jamaica, Poland or Hungary, and men and women born in Irish Republic. The largest increases occurred for those born in East Africa, West Africa or Pakistan.

**Table 1:** Trends in coronary heart disease death rates* per 100 000 in England and Wales, by country of birth among those aged 30–69 years

	1979–83			1989–93			1999–2003		
	Population	Deaths	Rate (95% CI)	Population	Deaths	Rate (95% CI)	Population	Deaths	Rate (95% CI)
*Men*									
England and Wales	9901394	183412	342.0 (340.4 to 343.5)	10401929	131505	240.0 (238.7 to 241.3)	11325598	74951	132.6 (131.6 to 133.5)
Jamaica	61402	397	154.2 (137.7 to 170.8)	59942	484	129.9 (117.8 to 141.9)	48674	402	107.4 (95.8 to 118.9)
Other Caribbean	39074	216	168.2 (142.6 to 193.7)	40752	274	121.3 (106.6 to 136.1)	35727	236	85.9 (74.2 to 97.7)
West Africa	17075	82	260.0 (195.8 to 324.3)	32103	111	155.3 (123.4 to 187.1)	66526	145	83.0 (68.7 to 97.2)
East Africa	33970	290	455.2 (381.9 to 528.5)	74278	467	311.6 (279.2 to 344.1)	99207	558	176.4 (160.6 to 192.2)
India	130003	2501	477.8 (458.8 to 496.8)	151155	2452	337.7 (324.3 to 351.0)	160662	1770	190.8 (181.8 to 199.7)
Pakistan	48551	641	390.7 (355.2 to 426.3)	74297	1002	363.7 (340.5 to 386.8)	102401	1017	255.3 (239.2 to 271.5)
Bangladesh	15909	248	466.3 (389.7 to 542.9)	25551	448	404.4 (361.5 to 447.3)	43305	474	279.3 (250.5 to 308.0)
Scotland	244362	5282	413.1 (401.9 to 424.3)	251316	3632	284.5 (275.2 to 293.8)	282524	2406	166.3 (159.7 to 173.0)
Northern Ireland	69108	1396	398.8 (377.9 to 419.7)	70120	1078	288.8 (271.5 to 306.1)	73068	627	160.0 (147.5 to 172.6)
Republic of Ireland	219663	4924	405.1 (393.8 to 416.4)	189829	3726	303.2 (293.4 to 313.0)	146708	2187	191.9 (183.4 to 200.4)
Italy	33737	283	231.3 (202.4 to 260.2)	33698	276	160.0 (140.9 to 179.0)	35183	226	106.2 (92.1 to 120.3)
Spain	12296	74	210.0 (159.3 to 260.7)	11101	73	159.7 (121.0 to 198.3)	11531	55	98.3 (72.1 to 124.4)
France	6299	92	289.3 (228.7 to 349.8)	8346	80	192.3 (148.5 to 236.1)	14828	48	140.3 (98.1 to 182.6)
Poland	45221	1814	400.3 (373.6 to 427.0)	23566	1047	301.4 (263.9 to 338.9)	6135	92	260.9 (201.8 to 319.9)
Hungary	7282	114	335.2 (270.4 to 400.0)	6085	158	339.4 (280.9 to 397.8)	4245	91	198.9 (120.8 to 277.0)
									
*Women*									
England and Wales	10368408	62363	97.8 (97.0 to 98.5)	10683031	47263	75.4 (74.7 to 76.1)	11582990	23464	39.2 (38.7 to 39.7)
Jamaica	58674	125	62.0 (49.6 to 74.3)	64537	217	65.8 (56.9 to 74.7)	59323	200	48.1 (41.1 to 55.1)
Other Caribbean	38572	66	68.2 (49.2 to 87.1)	45515	91	49.5 (38.8 to 60.1)	45102	125	41.3 (34.0 to 48.6)
East Africa	28631	43	122.7 (79.7 to 165.6)	66821	92	85.7 (66.6 to 104.8)	95959	140	56.4 (46.8 to 66.1)
India	118711	723	154.1 (142.9 to 165.4)	150548	875	128.9 (120.3 to 137.4)	168387	659	72.0 (66.5 to 77.5)
Pakistan	33013	73	111.1 (82.6 to 139.5)	65538	178	100.5 (84.8 to 116.2)	96584	326	95.9 (85.4 to 106.4)
Scotland	226881	1613	128.1 (121.8 to 134.4)	233274	1276	97.3 (91.8 to 102.8)	265548	742	54.5 (50.6 to 58.4)
Northern Ireland	68440	482	132.3 (120.5 to 144.2)	70638	383	96.4 (86.5 to 106.2)	75004	222	54.1 (47.0 to 61.3)
Republic of Ireland	237199	1579	117.3 (111.5 to 123.1)	208662	1299	90.8 (85.8 to 95.8)	168995	722	51.7 (47.7 to 55.6)
Italy	36712	117	83.5 (67.1 to 100.0)	34065	157	56.9 (47.7 to 66.0)	30167	89	37.0 (28.2 to 45.8)

*Rates are age adjusted to the Europe 2000 population.

### Mortality from coronary disease among men

**Figure 1:**
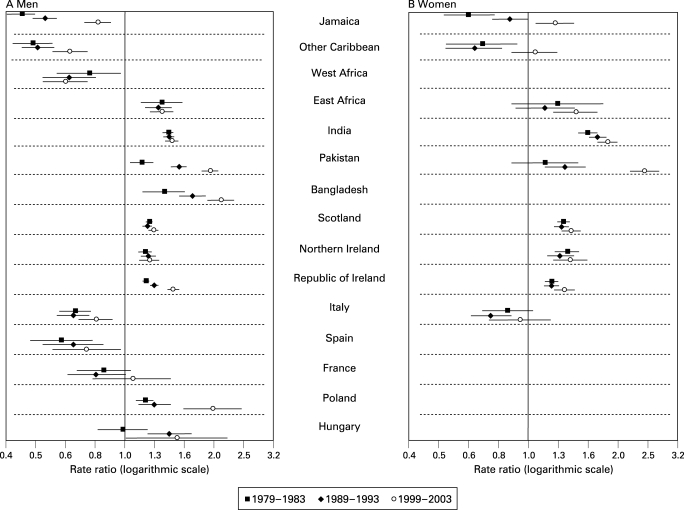
Trends in coronary heart disease mortality in England and Wales for selected migrant groups, aged 30–69 years: rate ratios and 95% confidence intervals (rates for those born in England and Wales as reference).

In all three time periods, compared with men born in England and Wales, men born in Jamaica, other Caribbean countries, West Africa, Italy or Spain had significantly lower coronary mortality, while those born in East Africa, India, Pakistan, Bangladesh, Scotland, Northern Ireland, Republic of Ireland or Poland had higher mortality (table 1 and fig 1A). The death rates for those born in Hungary were higher only in the last two periods. Increasing rate ratios for men born in Pakistan, Bangladesh, the Republic of Ireland or Poland reflect a widening of mortality differences relative to men born in England and Wales. For men born in Jamaica or other Caribbean countries, the increasing rate ratios reflected a shift towards the rates for men born in England and Wales.

**Figure 2:**
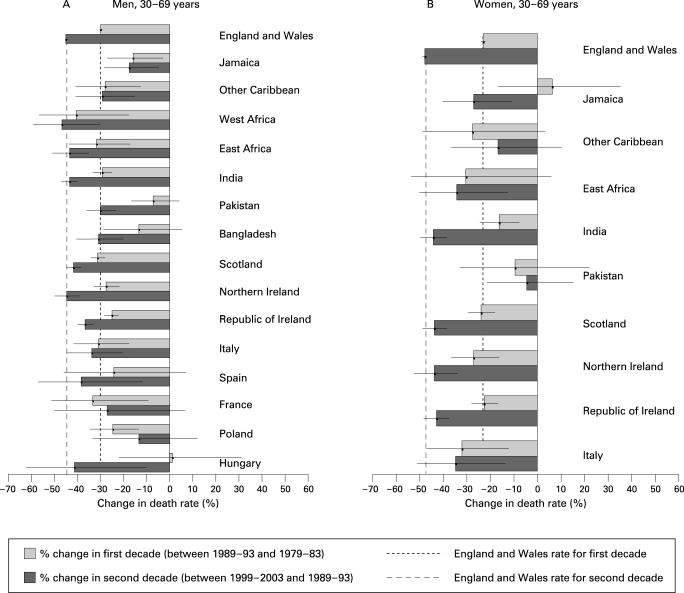
Percentage change in coronary heart disease death rates in England and Wales between 1989–93 and 1979–83, and between 1999–2003 and 1989–93.

With few exceptions, coronary death rates declined in the first and second decades (fig 2A), and there was a broad pattern of greater declines in the second than the first decade. The declines were not significant in the first decade for men from Pakistan, Bangladesh, Spain or Hungary and in the second decade for those from Poland. Compared with England and Wales in the second decade, there were significantly smaller declines for many migrant groups (Jamaica, other Caribbean, Pakistan, Bangladesh, Republic of Ireland and Poland), explaining the shifts in relative mortality described above.

### Mortality from coronary disease among women

Among women, consistently higher mortality was seen for those born in India, Scotland, Northern Ireland or Republic of Ireland in all time periods compared to women born in England and Wales (table 1 and fig 1B). In 1999–2003 women born in Jamaica had higher mortality for the first time and the excess was significant for Pakistan born women in the last two time periods. Rate ratios increased for these two groups, such that the rate for Pakistan-born in 1999–2003 was more than twice that of England and Wales born women.

Figure 2B shows that, as with men, declines were apparent for many groups, and generally greater in the second decade than the first. In the first decade the declines were not significant for women born in Jamaica, other Caribbean, East Africa or Pakistan and in the second decade for those born in other Caribbean or Pakistan. Compared with declines for women born in England and Wales, the decline was significantly smaller in the second decade for women from Jamaica, other Caribbean countries or Pakistan which explains the shift in rate ratios in fig 1B.

### Mortality from stroke among men

**Figure 3:**
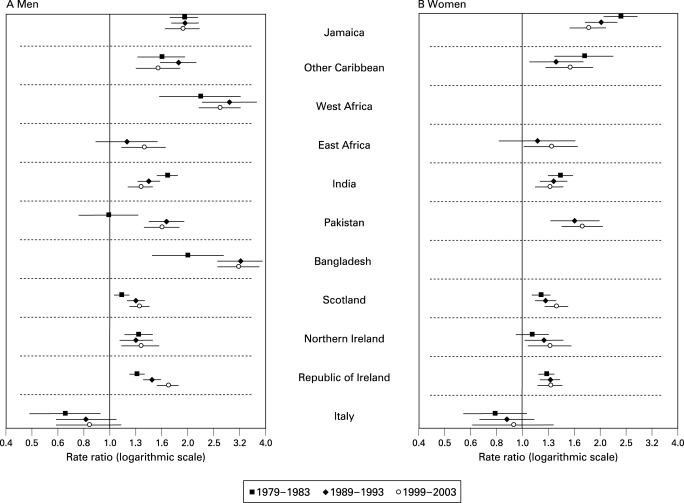
Trends in stroke mortality in England and Wales for selected migrant groups, aged 30–69 years: rate ratios and 95% confidence intervals (rates for those born in England and Wales as reference).

Apart from men from East Africa, Pakistan or Italy, stroke mortality was higher for migrant groups in each time period compared with men born in England and Wales (table 2 and fig 3A). The relatively lower mortality of men from Italy was significant only in 1979–83. In 1989–93 and 1999–2003, men born in West Africa or Bangladesh had the highest rates. Excess mortality reduced for men born in India, but for men born in Pakistan or Scotland the excess mortality increased in 1989–93 and remained static, and for men born in the Republic of Ireland excess mortality increased across the three periods (fig 3A).

**Table 2:** Trends in stroke death rates* per 100 000 in England and Wales, by country of birth among those aged 30–69 years

	1979–83		1989–93		1999–2003	
	Deaths	Rate (95% CI)	Deaths	Rate (95% CI)	Deaths	Rate (95% CI)
*Men*						
England and Wales	32465	58.8 (58.1 to 59.4)	21772	38.9 (38.4 to 39.5)	15256	26.8 (26.4 to 27.3)
Jamaica	280	113.3 (98.8 to 127.7)	286	75.9 (66.8 to 85.0)	204	51.0 (43.4 to 58.6)
Other Caribbean	112	92.7 (73.4 to 112.1)	155	71.4 (59.9 to 83.0)	115	40.9 (32.9 to 48.9)
West Africa	43	131.0 (83.9 to 178.1)	80	112.4 (85.2 to 139.6)	123	70.9 (57.6 to 84.2)
East Africa†			68	45.2 (32.7 to 57.8)	119	36.3 (29.2 to 43.4)
India	491	97.4 (88.7 to 106.2)	395	54.9 (49.5 to 60.4)	328	35.2 (31.4 to 39.1)
Pakistan	80	58.5 (43.2 to 73.8)	173	64.2 (54.3 to 74.1)	170	42.3 (35.7 to 48.9)
Bangladesh	62	117.1 (79.8 to 154.5)	130	123.8 (99.2 to 148.4)	147	83.5 (68.3 to 98.8)
Scotland	850	65.5 (61.1 to 69.9)	640	49.0 (45.2 to 52.9)	501	34.8 (31.7 to 37.8)
Northern Ireland	267	75.6 (66.5 to 84.7)	189	49.2 (42.1 to 56.2)	137	35.0 (29.1 to 40.9)
Republic of Ireland	910	74.5 (69.7 to 79.4)	700	56.4 (52.2 to 60.7)	501	44.8 (40.6 to 49.0)
Italy	43	39.5 (26.9 to 52.2)	54	31.5 (23.0 to 40.0)	48	22.3 (15.9 to 28.7)
						
*Women*						
England and Wales	28405	45.6 (45.1 to 46.2)	18120	29.6 (29.2 to 30.0)	12186	20.6 (20.2 to 21.0)
Jamaica	221	109.4 (93.0 to 125.8)	193	59.5 (51.0 to 68.1)	153	36.9 (30.8 to 43.0)
Other Caribbean	78	78.8 (58.4 to 99.2)	74	40.0 (30.4 to 49.6)	90	31.4 (24.9 to 37.9)
East Africa†			47	33.8 (22.4 to 45.2)	79	26.6 (20.2 to 32.9)
India	304	64.0 (56.8 to 71.3)	269	39.1 (34.4 to 43.8)	236	26.1 (22.8 to 29.5)
Pakistan†			93	47.1 (36.7 to 57.5)	124	35.0 (28.7 to 41.3)
Scotland	661	53.8 (49.7 to 58.0)	479	36.4 (33.0 to 39.7)	379	27.8 (25.0 to 30.6)
Northern Ireland	178	49.9 (42.5 to 57.3)	138	35.8 (29.7 to 41.9)	105	26.1 (21.1 to 31.1)
Republic of Ireland	750	56.7 (52.7 to 60.8)	523	37.9 (34.6 to 41.3)	349	26.3 (23.4 to 29.2)
Italy	56	35.5 (25.3 to 45.6)	70	25.8 (19.6 to 32.1)	40	19.0 (12.2 to 25.8)

*Rates are age adjusted to the Europe 2000 population.

†Blank cells as number of deaths <40.

**Figure 4:**
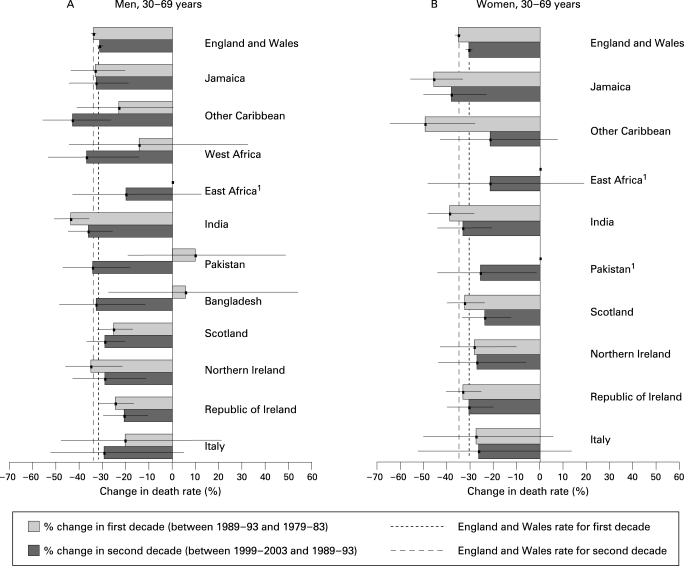
Percentage change in stroke death rates in England and Wales between 1989–93 and 1979–83, and between 1999–2003 and 1989–93.^1^ Missing for the first decade as <10 deaths in each 10-year age group in 1979–83.

Figure 4A shows a pattern of declines across the groups, though not significant in the first decade for men from other Caribbean countries, West Africa, Pakistan, Bangladesh or Italy, or in the second decade for East Africa. Compared with men born in England and Wales, men born in India benefited from larger declines in the first decade. Significantly smaller declines were seen for men from Scotland in the first decade and for those from the Republic of Ireland in both decades.

### Mortality from stroke among women

Death rates for stroke remained higher among most migrant groups compared with women born in England and Wales (table 2 and fig 3B). As with men from Italy, the rates for women were exceptions as they were not significantly different. For other groups the rate ratios remained generally static (fig 3B). Stroke rates declined across most groups in both decades and the size of the declines did not vary significantly across the groups (fig 4B).

## DISCUSSION

The substantial declines in coronary and stroke death rates for those born in England and Wales are similar to those in other Western European countries and are consistent with favourable long-term changes in population risk factors (smoking, cholesterol, blood pressure) and in treatment.^8^ ^9^ Few data on risk factor trends for migrant groups are available, but the results here correspond with a gradual convergence of risk for some groups towards that of England and Wales. The relative impact of risk factors such as obesity and diabetes may differ across migrant groups but generally smaller declines compared with those born in England and Wales suggest a lag in behaviour modification or differential access to quality care, or both.

The Health Survey for England^10^ is the only national data source of risk factor exposures for ethnic groups. Trends for smoking provide some evidence of an emergent harmful exposure influencing cardiovascular risk in both men and women from the Caribbean and men of Pakistani and Bangladeshi origin. In 2004 smoking prevalence among Black Caribbean men and women was similar to that of the general population, while for Pakistani and Bangladeshi men it was higher. The proportion of ex-regular smokers in these groups was lower and of those who never smoked was higher. Local studies suggest that the prevalence of smoking in Caribbeans was previously low,^5^ ^11^ so a rise in smoking is plausible. A rise can also be expected for South Asians as findings from the Stockport Stroke Risk Factor Screening Programme showed an increase in smoking prevalence in South Asians between 1988 and 1999.^12^

Heterogeneity of risk in South Asians is known.^13^ ^14^ Our analyses suggest that the lack of decline observed for South Asian migrants in the earlier work^2^ was largely due to the trends for Pakistani and Bangladeshi migrants. There are no data on long-term trends in risk factors for the individual South Asian groups but the data from the Health Survey for England^10^ suggest that trends in mortality may continue to be relatively more adverse for Bangladeshis and Pakistanis than Indians. Between 1999 and 2004, the prevalence of cardiovascular disease in Pakistanis doubled, and among women aged 55 years and older, the prevalence of diabetes was more than fivefold that of all women and increased by 16% (compared with 2% for all women). The prevalence of overweight including obesity in Bangladeshis, though lower than for the general population, increased between 1999 and 2004 by 8% for men and 14% for women (compared with 4% for all men and 3% for all women) and the prevalence of diabetes was highest in Bangladeshi men, fourfold that of men in general, in both years. 

There was a large rise in Bangladeshi migrants (70% for men) between the 1991 and 2001 censuses compared with Pakistani (38% for men, 47% for women) or Indian (6% for men, 12% for women) migrants, suggesting that exposures in home countries might be relatively more important for Bangladeshis. In the INTERHEART study of South Asians in South Asian countries, Bangladeshis had the highest prevalence of most risk factors compared with other South Asians.^6^ The size of the Caribbean population reduced for men and changed little for women, suggesting less influx of new migrants so that acculturation to UK lifestyles may have a role. Other work signals that a change in coronary risk among Caribbeans can be expected, with a shift away from traditional diets with high fresh fruit and vegetable content^15^ and a lowering of the level of protective high-density cholesterol.^16^ International comparative data also provide supporting evidence for a faster transition in coronary risk for Caribbeans than West Africans. The prevalence of diabetes^17^ and hypertension^18^ in populations of African origin living in the Caribbean is intermediate between those of Africans in Africa and in Britain.

The impact of differential access to health care on ethnic differences in cardiovascular mortality is unclear. South Asians are more likely to seek care but the quality of medical management has been questioned.^19^ South Asians are less likely than White British to present with classic symptoms of myocardial infarction, and some argue that this makes diagnosis difficult and possibly delays essential treatment. Bangladeshis and Pakistanis appear to fare worse than Indians as they are less likely to have invasive management of coronary disease.^20^ ^21^ Declining case fatality rates among South Asians, however, for acute myocardial infarction between 1998 and 2002 and reduction in infarct severity suggest improvement in survival and correspond with the data reported for the second decade.^22^ In the Health Survey for England 2004,^10^ the prevalence of high blood pressure was greater than average only among Black Caribbean, Black African and Bangladeshi women, suggesting that the consistent excess stroke mortality for most groups is linked to differences in access to healthcare. The evidence suggests that although detection and control of hypertension may have improved, ethnic differences remain.^10^ ^11^

The results for Southern/Eastern European and French migrants are partially consistent with rates in home countries, suggesting some retention of risk. Coronary mortality is lower in Italy, France and Spain than the United Kingdom and higher (though declining) in Poland (since the late 1990s) and Hungary (since the late 1980s).^23^ Stroke mortality among men in Poland and Hungary is more than twice that of the United Kingdom. The cause of low coronary mortality in Southern European countries and France continues to be debated, but is generally thought to be linked to high intake of monosaturated fats and antioxidants in Southern Europe.^24^ Whereas men from Italy or Spain retained lower coronary mortality than men born in England and Wales, the rates for men from France were not significantly different, which raises the question of whether retention of traditional behavioural practices differs across the groups. Higher cardiovascular mortality in Eastern Europe has been linked to higher levels of smoking, alcohol consumption and saturated fat intake.^25^

Greater socioeconomic disadvantage among minorities may contribute to smaller declines in mortality. Persisting disadvantage is more common among South Asian and Caribbean migrants, and downward mobility is associated with more ill health among Caribbeans.^26^ Research on the impact of disadvantage on ethnic differences in cardiovascular health is limited^13^ (and non-existent for Southern or Eastern European migrants). The evidence suggests that higher disadvantage does not fully explain the patterns of excess mortality observed.^4^

Our results are subject to the usual limitations of cross-sectional data—notably, misclassification of country of birth between the census and death certificates, and selection bias (health status on migration). This is most pertinent for older South Asians. Pakistanis and Bangladeshis born before the formation of Pakistan (1947) and Bangladesh (1971) may have recorded India as country of birth in the census but relatives may have reported Pakistan or Bangladesh at death. Temporal trends might be influenced by cohort trends—age at entry to the UK and duration of exposure to the UK environment all being potential confounders.^27^ Stratification by age provides some clues of cohort effects if we assume that older migrants have longer duration of residence. These data were not included owing to space restrictions. The trends were largely consistent at ages 45–59 and 60–69 years. Migration flows may also affect cross-sectional analyses of mortality trends. New migrants may have increased mortality risks owing to low uptake of healthcare, and people at high cardiovascular risk might have migrated.

Reliable national trend data from West Africa and the Indian subcontinent are lacking, but local studies suggest that cardiovascular risk, though changing, remains lower in comparison with British data. Most West Africans in Britain are from Ghana and Nigeria where hypertension prevalence is lower than for Africans in the UK.^28^ ^29^ The Caribbean, Polish and Hungarian populations are least likely to be affected by an influx of new migrants. Ending right of entry from British colonies to the UK after the 1962, the Commonwealth Act largely stemmed the Caribbean inflow. Before 2004, Hungarian and Polish migrants arrived mainly after the second world war. The longstanding debate on whether the high mortality associated with Scottish and Irish migrants is related to inflows of ill-fit individuals (linked to ease of migration) may be relevant, though the high mortality of second and third generations of Irish born in the UK^30^ challenges this as the sole explanation. ICD10 assigned more deaths to stroke than ICD9, but this is unlikely to affect differences in trends by country of birth groupings as this bias should affect all groups equally.

## CONCLUSION

There was a general pattern of decline in death rates between 1979 and 2003, but the declines were smaller for many migrant groups than for people born in England and Wales. As a result, the groups with mortality higher than people born in England and Wales remained consistently so over the period and for some, particularly in the case of coronary mortality for men born in Pakistan, Bangladesh and Poland, the disparity increased. A striking feature of these trends is the erosion of low coronary disease mortality in some groups. Women from Jamaica and men from Hungary now have coronary death rates higher than those born in England and Wales. Modifiable risk factors such as smoking, obesity and lack of sufficient physical activity are related to cardiovascular risk in all of these groups. The large number of countries included in this analysis reflects the increasing social complexity of the UK and a challenge for public health to deliver maximum coverage of prevention and treatment programmes across all groups.
